# The role of financial strain and self-control in explaining health behaviours: the GLOBE study

**DOI:** 10.1093/eurpub/ckx212

**Published:** 2017-12-08

**Authors:** Mariëlle A Beenackers, Joost Oude Groeniger, Frank J van Lenthe, Carlijn B M Kamphuis

**Affiliations:** 1 Department of Public Health, Erasmus University Medical Centre, Rotterdam, The Netherlands; 2 Department of Human Geography and Spatial Planning, Utrecht University, Utrecht, The Netherlands

## Abstract

**Background:**

Why lower socioeconomic groups behave less healthily can only partly be explained by direct costs of behaving healthily. We hypothesize that low income increases the risk of facing financial strain. Experiencing financial strain takes up cognitive ‘bandwidth’ and leads to less self-control, and subsequently results in more unhealthy behaviour. We therefore aim to investigate (i) whether a low income increases the likelihood of experiencing financial strain and of unhealthy behaviours, (ii) to what extent more financial strain is associated with less self-control and, subsequently, (iii) whether less self-control is related to more unhealthy behaviour.

**Methods:**

Cross-sectional survey data were obtained from participants (25–75 years) in the fifth wave of the Dutch GLOBE study (*N* = 2812) in 2014. The associations between income, financial strain, self-control and health-behaviour-related outcomes (physical inactivity in leisure-time, obesity, smoking, excessive alcohol intake, and weekly fruit and vegetable intake) were analysed with linear regression and generalized linear regression models (log link).

**Results:**

Experiencing great compared with no financial strain increased the risk of all health-behaviour-related outcomes, independent of income. Low self-control, as compared with high self-control, also increased the risk of an unhealthy lifestyle. Taking self-control into account slightly attenuated the associations between financial strain and the outcomes.

**Conclusion:**

Great financial strain and low self-control are consistently associated with unhealthy behaviours. Self-control may partly mediate between financial strain and unhealthy behaviour. Interventions that relieve financial strain may free up cognitive bandwidth and improve health behaviour.

## Introduction

Socioeconomic health inequalities are an important societal challenge.^1^^,^^2^ Unhealthy behaviours, such as smoking and physical inactivity, explain a large part of these inequalities since low socioeconomic groups generally act more unhealthily.^3–5^ Partly, this may be attributed to lower socioeconomic groups often having a lower disposable income, which may be a barrier for purchasing goods or services that are needed for behaving healthily (e.g. sports equipment). However, smoking is more prevalent in lower socioeconomic groups but actually costs money, while recreational walking is more prevalent in higher socioeconomic groups and free of costs. Therefore, other mechanisms through which poor material circumstances contribute to inequalities in health behaviours must also play a role.

Poorer material circumstances can co-occur with financial strain: i.e. having difficulties making ends meet, and paying bills for basic needs such as food, housing, and electricity. Financial strain is a constant stressor that forces daily difficult financial decision making on basic matters such as food and clothing. This relentless stress and feeling of lack of control negatively impacts health.^6^^,^^7^ The ‘scarcity theory’^8^^,^^9^ suggests that dealing with scarcity (such as scarcity of money) takes up ‘cognitive bandwidth’, i.e. ‘our computational capacity, our ability to pay attention, to make good decisions, to stick with our plans, and to resist temptations’ (pp. 41–42).^9^ An important pathway through which a reduced cognitive bandwidth may impede a healthy lifestyle is via self-control. Self-control is regarded as the capacity to ‘regulate cognition and behaviour in order to achieve long-term goals’.^10^ Self-control is a limited resource and can be depleted when demands are high.^9^^,^^10^ Therefore, dealing with a scarcity of money may tax one’s level of self-control, which leaves little self-control aside for making healthy life choices. Self-control is much needed for making healthy choices in the current obesogenic environment. In these environments, the unhealthy choice (i.e. sedentary behaviour, unhealthy food choices) is often the easier choice. Further, when self-control is low, stress may more easily trigger unhealthy coping responses such as smoking and excessive alcohol consumption, and it is more difficult to resist social pressure and unhealthy social modelling steering towards an unhealthy lifestyle. Lower socioeconomic groups are more often exposed to these unfavourable circumstances and are also more likely to experience financial strain. This combination places large demands on self-control with respect to health behaviours.

Another implicit assumption that underlies this line of reasoning is that health-behavioural decisions are largely made unconsciously. Behavioural change theories that have dominated for decades, such as the theory of planned behaviour,^11^ suggest health behaviours to come forth from rational choices. However, theories originating in psychology and behavioural economics suggest that many of our choices are irrational, impulsive and automatic rather than rational.^12^ When self-control is low (i.e. depleted due to dealing with financial difficulties) or temptations are large (as in the obesogenic environment), the impulsive responses take over more easily, which more likely leads to unhealthy rather than healthy behaviours.

In line with these theories, we hypothesize that constant financial strain takes up cognitive bandwidth which leaves less cognitive bandwidth to exert self-control in other aspects in life such as making healthy behavioural choices. Therefore, our objectives are to investigate (i) whether a low income increases the likelihood of experiencing financial strain and of unhealthy behaviours, (ii) to what extent more financial strain is associated with less self-control and, subsequently, (iii) whether less self-control is related to more unhealthy behaviour.

## Methods

### Data

Data were collected by means of a large-scale postal survey within the 2014 survey of the Dutch population-based GLOBE study (response = 45.5%). A cross-sectional sample of participants (25–75 years) living in Eindhoven and surrounding cities was used in the analyses (*N* = 2812). More detailed information on the objectives, study design and data collection of the Dutch GLOBE study can be found elsewhere.^13–15^ The use of personal data in the GLOBE study is in compliance with the Dutch Personal Data Protection Act and the Municipal Database Act, and has been registered with the Dutch Data Protection Authority (number 1248943).

### Measures

#### Income

Household equivalent income was measured as the level of monthly household income divided by the square root of the number of people living from this income. Household equivalent income was subsequently divided into quartiles.

#### Financial strain

Financial strain was assessed by two questions addressing (i) whether participants could make ends meet considering their monthly household income and (ii) whether they had experienced any financial difficulties in paying bills for food, rent, electricity and so forth during the preceding year. The combined measure of financial strain considered participants to have ‘no financial strain’ if they could make ends meet fairly easy or easy or if they experienced no financial difficulties in the preceding year. Participants were considered to have ‘some financial strain’ if they could make ends meet with some difficulty or if they experienced some financial difficulties in the preceding year. Participants were considered to have ‘great financial strain’ if they had great difficulty making ends meet or if they experienced large financial difficulties in the preceding year.

#### Self-control

Self-control was measured using the Brief Self-Control Scale by Tangney et al.^16^ The scale consists of 13 items which were rated on a 5-point scale anchored from (1) not at all like me to (5) very much like me (potential range of the scale: 13–65).

#### Health-behaviour-related outcomes

Physical inactivity during leisure time was measured using the validated Short QUestionnaire to ASsess Health-enhancing physical activity (SQUASH).^17^ The measure was highly skewed with many respondents not being active at all. Participants were considered to be physically inactive if they were active less than once per week for 30 minutes or more at moderate intensity (moderate intensity = 4–6 MET for 18–55 years and 3–5 MET for 55+ years). Those who were active during leisure time were considered the reference category.

Although obesity is not a health behaviour, we included this outcome in the analyses as an indicator of an unhealthy balance between diet and physical activity. Body mass index (BMI) was calculated by self-reported height and weight. Respondents with a BMI of 30 or higher were categorized as being obese. Having a BMI of below 30 was used as the reference category.

Current daily smokers were identified by the question ‘Do you smoke?’. This includes smokers of cigarettes, pipes, cigars and e-cigarettes. All non-smokers, former smokers and occasional smokers were grouped into the reference category.

Excessive alcohol intake was measured by asking participants how often they consumed alcoholic beverages and if so, how many alcoholic beverages they consumed on a drinking day. Participants were considered to have an excessive alcohol intake if they consumed over 14 (males) or 7 (females) alcoholic beverages a week or over 6 (male) or 4 (female) beverages a day on the day they drank alcohol. Non-excessive drinking behaviour was used as the reference category.

Fruit and vegetable intake was assessed with a food frequency questionnaire. Participants reported their weekly frequency of fruit and vegetables intake in the previous month and the number of portions they consumed on a typical occasion. Portions were defined as one unit of fruit (e.g. one banana, a small bowl of grapes) or one serving spoon of vegetables (=50 g). Total weekly intake of fruit and vegetables (in 100 g) was calculated by using the two questions above and by defining one piece of fruit to be equivalent to 100 g.

#### Confounders

Potential confounders included were age, gender (male, female), highest educational level based on ISCED 2011 categories [low (ISCED 0-2), medium (ISCED 3-4), high (ISCED 5-8)], living together with a partner (yes, no), having children living at home (yes, no), country of birth (Netherlands, other), and employment status [employed, unemployed, retired, non-employed (students, homemaker)].

### Statistical analysis

Studying mediation for dichotomous outcomes poses several challenges.^18^ Firstly, when the outcome is common (>10%), such as in our study, the odds ratios in a standard logistic regression no longer resemble the risk ratios and problems of non-collapsibility arise. To tackle this problem, we used generalized linear models with a log-link function to study the associations between household equivalent income, financial strain, self-control and the dichotomous outcomes physical inactivity, obesity, daily smoking and excessive alcohol intake.^18^^,^^19^ These models produce risk ratios which do not have the problem of non-collapsibility. Secondly, in mediation models, no exposure–mediator interaction should exist. This assumption was checked and not violated in our study. Linear regression models were used for fruit and vegetable intake (in units of 100 g/week).

Four models were constructed for each health-behaviour-related outcome. The first model contained only household equivalent income, in order to investigate income inequalities in the health-behaviour-related outcomes. The second model was similar to the first one but adjusted for all confounders. Financial strain was added to the third model. In the fourth and final model, we additionally included self-control. The mediating role of self-control in the association between financial strain and health behaviour was checked by calculating the percentage change in risk ratios (loglinear models) or betas (linear models) between models 3 and 4. A bootstrapping procedure was used to calculate a 95% confidence interval (CI) around the percentage change. The association between financial strain and self-control was studied via linear regression adjusting for all confounders. The variation inflation factor was examined to check for collinearity, especially between the socioeconomic indicators, but no strong collinearity was detected [1.03, 2.58].

Overall, missing values of questionnaire items varied from <1% to 3.3% per item, with only income having 12.7% missing values. Missing data were handled using multiple imputations (*m* = 5). Respondents with missing values on an outcome variable were excluded from all analyses with that particular outcome.

All analyses were weighted by respondent-level sample weights to account for the sampling strategy used within the GLOBE study. All regression analyses were carried out in STATA 14.1 (StataCorp LP, College Station, TX). The bootstrapping procedures were carried out in R (version 3.3.3).

## Results

Over two-thirds of the respondents (68.1%) did not experience any financial strain while 6.9% reported great financial strain (table 1). Within those experiencing great financial strain, most had low household equivalent income (77.7%) compared with high (2.7%).

**Table 1:** Description of the sample by financial strain (*n* = 2812)

	Total	Financial strain (1.8% missing)			Self-control (3.3% missing)
		No strain (68.1%)	Some strain (25.0%)	Great strain (6.9%)	
**Demographics**					
Gender (no missing)					
Men	44.8%	46.5	43.3	33.3	44.0 ± 6.8
Women	55.2%	53.5	56.6	66.6	44.2 ± 6.9
Age groups (mean ± SD) (no missing)	48.8 ± 14.9	49.0 ± 15.0	48.2 ± 14.4	48.6 ± 14.9	–
25–34 years	25.6%	26.2	23.9	23.4	42.4 ± 7.0
35–44 years	16.9%	16.4	18.7	17.0	43.9 ± 7.8
45–54 years	17.6%	15.7	21.5	23.6	44.3 ± 6.2
55–64 years	19.3%	19.8	18.0	16.9	44.9 ± 5.8
65–74 years	20.7%	22.0	17.9	19.1	45.4 ± 6.8
Education (0.9% missing)					
Low (ISCED 0–2)	25.8%	21.0	33.7	43.7	44.6 ± 7.0
Medium (ISCED 3–4)	25.1%	21.2	33.2	30.9	43.5 ± 6.9
High (ISCED 5–8)	49.1%	57.8	33.2	25.4	44.2 ± 6.7
Living together (1.1% missing)					
No, does not live together with partner	26.0%	20.2	33.5	52.8	42.6 ± 7.1
Yes, lives together with partner	74.0%	79.8	66.5	47.2	44.6 ± 6.7
Country of birth (0.5% missing)					
Netherlands	88.5%	91.7	85.1	74.3	44.0 ± 6.8
Outside of the Netherlands	11.5%	8.3	14.9	25.7	45.2 ± 7.3
Children living at home (no missing)					
No, no children living at home	64.3%	67.1	59.2	56.0	44.0 ± 6.9
Yes, children living at home	35.7%	32.9	40.8	44.0	44.4 ± 6.8
Employment status (1.9% missing)					
Employed	63.6%	67.1	59.0	44.8	44.0 ± 6.7
Retired	20.4%	21.9	17.9	17.8	45.3 ± 6.7
Unemployed	8.0%	4.5	12.6	24.9	42.7 ± 7.4
Non-employed (students, housewives)	7.9%	6.5	10.4	12.6	43.7 ± 7.2
Household equivalent income quartiles (12.7% missing)					
Lowest	25.7%	12.6	47.1	77.7	43.5 ± 7.4
Middle low	27.5%	27.0	32.6	14.2	43.9 ± 6.8
Middle high	28.5%	35.7	15.5	5.4	44.1 ± 6.6
Highest	18.2%	24.7	4.8	2.7	44.6 ± 6.8
**Self-control (mean** ± **SD)** (3.3% missing)	44.1 ± 6.9	44.7 ± 6.7	42.8 ± 7.0	42.3 ± 7.4	–
**Health-behaviour-related measures**					
Physically inactive during leisure time (1.4% missing)					
Active	88.8%	90.8	86.1	79.7	44.3 ± 6.8
Inactive	11.2%	9.2	13.9	20.3	42.2 ± 7.1
Obese (1.4% missing)					
Not obese	86.4%	89.0	81.8	75.6	44.5 ± 6.8
Obese	13.6%	11.0	18.2	24.4	41.6 ± 6.8
Daily smokers (0.7% missing)					
Non-smoker or occasional smoker	84.5%	88.8	78.4	64.2	44.5 ± 6.7
Daily smoker	15.5%	11.2	21.6	35.8	41.7 ± 7.0
Excessive alcohol intake (2.6% missing)					
No excessive alcohol intake	80.1%	80.5	78.8	79.1	44.5 ± 6.8
Excessive alcohol intake	19.9%	19.5	21.2	20.9	42.4 ± 6.9
Weekly intake of fruit and vegetables (×100 g) (mean ± SD) (3.3% missing)	19.7 ± 10.0	20.9 ± 9.8	17.4 ± 9.6	16.9 ± 10.1	–
< –1 SD	16.7%	13.6%	67.4%	19.0%	42.2 ± 6.8
Mean ± 1SD	64.5%	22.9%	61.5%	15.6%	44.2 ± 6.8
> +1 SD	18.7%	24.4%	56.3%	19.3%	45.6 ± 6.6

Notes: Data in this table are weighted according to the sampling strategy. The data are not imputed. SD, standard deviation.

Lower household equivalent income was associated with a higher risk of leisure time physical inactivity, obesity, daily smoking and a lower fruit and vegetable intake in the crude models (Model 1, tables 2 and 3). However, the association was greatly reduced and no longer showed a clear gradient for most health behaviours when adjusted for educational level and other confounders (Model 2, tables 2 and 3).

**Table 2:** Generalized linear models with log link function for leisure time physical inactivity (*n* = 2772), obesity (*n* = 2772), daily smoking (*n* = 2791) and excessive alcohol intake (*n* = 2738)

	Model 1: Household income		Model 2: + confounders		Model 3: + financial strain		Model 4: + self-control		Percentage reduction in risk ratio from model 3 to model 4	
	RR^a^	95%CI^b^	RR	95%CI	RR	95%CI	RR	95%CI	%	95%CI
Leisure-time physical inactivity										
Household equivalent income										
Highest quartile	1.00		1.00		1.00		1.00			
Middle high quartile	1.53	0.98; 2.39	1.42	0.90; 2.45	1.42	0.90; 2.23	1.40	0.90; 2.20		
Middle low quartile	1.70	1.07; 2.71	1.39	0.84; 2.29	1.35	0.82; 2.22	1.35	0.82; 2.21		
Lowest quartile	2.05	1.35; 3.12	1.29	0.80; 2.10	1.17	0.71; 1.93	1.17	0.72; 1.92		
Financial strain										
No strain					1.00		1.00			
Some strain					1.14	0.86; 1.50	1.08	0.83; 1.42	–40%	–406%; 318%
Great strain					1.48	1.02; 2.14	1.37	0.95; 1.97	–23%	–103%; –2%
Self-control							0.97	0.95; 0.99		
Obesity										
Household equivalent income										
Highest quartile	1.00		1.00		1.00		1.00			
Middle high quartile	1.76	1.07; 2.89	1.49	0.90; 2.47	1.46	0.89; 2.42	1.48	0.90; 2.41		
Middle low quartile	2.15	1.30; 3.55	1.47	0.86; 2.52	1.38	0.80; 2.36	1.40	0.83; 2.37		
Lowest quartile	2.88	1.78; 4.67	1.79	1.04; 3.08	1.49	0.84; 2.63	1.53	0.89; 2.64		
Financial strain										
No strain					1.00		1.00			
Some strain					1.38	1.07; 1.77	1.26	0.99; 1.60	–32%	–102%; –13%
Great strain					1.64	1.17; 2.31	1.46	1.04; 2.06	–28%	–80%; –7%
Self-control							0.94	0.93; 0.96		
Daily smoking										
Household equivalent income										
Highest quartile	1.00		1.00		1.00		1.00			
Middle high quartile	1.30	0.88; 1.91	1.00	0.69; 1.47	0.99	0.68; 1.44	0.99	0.68; 1.44		
Middle low quartile	1.72	1.18; 2.51	1.11	0.74; 1.66	1.02	0.68; 1.54	1.02	0.68; 1.55		
Lowest quartile	2.58	1.81; 3.68	1.38	0.93; 2.04	1.10	0.72; 1.68	1.10	0.72; 1.70		
Financial strain										
No strain					1.00		1.00			
Some strain					1.44	1.15; 1.80	1.35	1.08; 1.69	–20%	–51%; –8%
Great strain					1.92	1.41; 2.62	1.78	1.32; 2.40	–16%	–31%; –5%
Self-control							0.96	0.95; 0.98		
Excessive alcohol intake										
Household equivalent income										
Highest quartile	1.00		1.00		1.00		1.00			
Middle high quartile	1.16	0.91; 1.49	1.05	0.82; 1.34	1.03	0.80; 1.32	1.03	0.80; 1.31		
Middle low quartile	1.02	0.78; 1.34	0.91	0.69; 1.21	0.86	0.64; 1.15	0.86	0.64; 1.16		
Lowest quartile	0.89	0.68; 1.17	0.84	0.61; 1.16	0.73	0.52; 1.03	0.75	0.53; 1.05		
Financial strain										
No strain					1.00		1.00			
Some strain					1.29	1.04; 1.59	1.19	0.97; 1.47	–33%	–141%; –13%
Great strain					1.41	0.99; 2.01	1.28	0.90; 1.81	–32%	–190%; 69%
Self-control							0.96	0.95; 0.98		

a RR = risk ratio.

b CI = confidence interval.

**Table 3:** Linear regression model for fruit and vegetable intake (*n* = 2720)

	Model 1: Household income		Model 2: + confounders		Model 3: + financial strain		Model 4: + self-control		Percentage reduction in beta from model 3 to model 4	
	*b*	95%CI^a^	*b*	95%CI	*b*	95%CI	*b*	95%CI	%	95%CI
Household equivalent income										
Highest quartile	Ref^b^		Ref		Ref		Ref			
Middle high quartile	–0.40	–1.65; 0.85	–0.20	–1.48; 1.07	–0.05	–1.32; 1.22	–0.01	–1.26; 1.25		
Middle low quartile	–1.03	–2.37; 0.32	–0.24	–1.68; 1.19	0.33	–1.14; 1.79	0.38	–1.06; 1.82		
Lowest quartile	–3.06	–4.32; –1.81	–1.76	–3.29; –0.23	–0.39	–2.01; 1.23	–0.43	–2.02; 1.16		
Financial strain										
No strain					Ref		Ref			
Some strain					–2.79	–3.83; –1.75	–2.43	–3.46; –1.40	–13%	–23%; –7%
Great strain					–3.11	–4.78; –1.44	–2.67	–4.31; –1.03	–14%	–33%; –6%
Self-control							0.19	0.13; 0.25		

a CI = confidence interval.

b Ref = reference.

Experiencing financial strain was associated with an increased risk of behaving unhealthily, independent of household equivalent income and other confounders (Model 3, tables 2 and 3). Financial strain was also associated with self-control (some strain *β* = –1.84, 95%CI: –2.58; –1.10 and great strain *β* = –2.38, 95%CI: −3.77; –1.00) in a linear regression model adjusted for household equivalent income, educational level, and all other confounders.

Self-control was associated with all health-behaviour-related outcomes in the fully adjusted models (Model 4, tables 2 and 3). When self-control was added to the models, the associations between experiencing great financial strain and the health-behaviour-related outcomes attenuated with 14–32% (last columns, tables 2 and 3).

## Discussion

Income inequalities were found for physical inactivity, obesity, smoking and fruit and vegetable intake, although these inequalities were greatly attenuated after adjustment for confounders. Experiencing financial strain and having low self-control both increased the risk of all health-behaviour-related outcomes. The associations between financial strain and unhealthy behaviours slightly attenuated when self-control was taken into account.

In line with previous research, we found income inequalities for all health behaviour outcomes,^20–23^ except excessive alcohol consumption.^24^ However, when adjusted for relevant confounders, the income inequalities were highly reduced and in most cases no longer showed a clear gradient. Further exploration revealed that especially educational level acted as a strong confounder. This has also been reported before in models where multiple socioeconomic indicators were included.^25^ This may imply that education-related resources (e.g. knowledge) are more important for healthy behaviours than income-related resources (e.g. money).

Those experiencing great financial strain appeared to have an increased risk of behaving unhealthily in our sample, independent of their income level. This suggests that it is not just the level of household equivalent income that is important for a healthy lifestyle, but whether this income is sufficient to make ends meet. Whether income is sufficient may depend on factors such as actual living costs (rent, mortgage), previous debts, perception (need to own high status luxury goods, etc.) and the social and cultural environment.

Previous research into the association of financial strain with health-related behaviours has focused mostly on maladaptive coping responses to stress such as tobacco and alcohol consumption. Several studies^26^^,^^27^ confirm our findings that financial stress increases alcohol and tobacco use. However, there is also evidence that these behaviours contribute to financial difficulties due to the costs involved with these behaviours.^28^ Financial stress has also been linked to weight gain and obesity which is in concordance with our results.^29^^,^^30^ In contrast to our findings, a recent Dutch study found that financial strain was associated with less good health but that had no (smoking and overweight) or only limited (heavy drinking) influence on health behaviours.^31^ Although there are studies about differences in physical activity and fruit and vegetable intake between different income groups,^21^^,^^23^ the evidence for the link between financial strain and these outcomes is scarce.

Our study supports the finding that low self-control increases the likelihood of an unhealthy lifestyle.^16^^,^^32–34^ The consistent association between self-control and an unhealthy lifestyle indicates that those who have higher levels of self-control are more capable of resisting impulses that may lead to unhealthy behaviours. Especially in an environment in which the unhealthy choice is often the default choice, demands for self-control are high. Additional analyses (results not shown) indicate that especially for smoking, alcohol consumption and BMI, there may be a dose–response association.

Further, the association between financial strain and a healthy lifestyle seems to be partly mediated by self-control. The scarcity theory suggests that financial strain may take up a large amount of cognitive bandwidth; a scarce resource.^8^^,^^9^ At the same time, behaving healthily demands high levels of self-control, also taking up cognitive bandwidth. Due to the scarcity of cognitive resources, these processes compete; when cognitive bandwidth is already engaged to deal with daily financial stress, there will be fewer resources available for self-control in behavioural choices. However, the mediation was only partial and limited in size indicating that there are other mechanisms (e.g. via stress, coping, sleep or locus of control) that may explain why experiencing financial hardship makes it more likely to behave unhealthily.

### Methodological considerations

This study is the first to look at income, financial strain as well as self-control for a broad spectrum of important health-behaviour-related outcomes. Some limitations and methodological reflections are in place for the interpretation of the findings of this study. First, the cross-sectional design restricts interpretation on causality and direction of the associations. This is particularly important since our hypothesis suggest temporality in which financial strain drains self-control and in turn leads to unhealthy behaviours. An alternative explanation of our findings is that self-control confounds the association between financial strain and an unhealthy lifestyle. Self-control as a disposition or trait may cause better financial management which could lead to less financial strain, and healthier behaviours. Additionally, some studies show that unhealthy behaviours that involve high costs such as smoking and alcohol consumption may deteriorate financial stress.^28^ Secondly, we used a measure of trait self-control developed by Tangney et al.^16^ This measure may not be very sensitive to depletion of self-control as suggested by the strength model.^10^ However, our results do suggest partial mediation by self-control and may therefore be sensitive enough to detect differences in self-control that are relevant for this research. Complex longitudinal designs including long-term momentary assessments of state self-control, and health behaviours may provide more causal insight into this mechanism. Furthermore, our health-behaviour-related outcomes were all self-reported which may have caused some misclassification. Lower socioeconomic groups are more susceptible for misreporting and therefore misclassification of being at risk.^35^ The socioeconomic inequalities may therefore be underestimated in our study. Due to the use of validated measures such as the SQUASH^17^ and the robustness of our, mainly dichotomous, outcomes we expect limited bias due to misclassification. Whereas the prevalence of health-related behaviours reported in our study is representative of the native Dutch population, it is likely less generalizable to ethnic minority groups since participants from non-Dutch origin are underrepresented in the GLOBE study.^14^

### Implications for public health research and practice

Our results imply that interventions aimed at relieving financial strain may improve health behaviours. Since the association between financial strain and a healthy lifestyle was independent of income, this may not solely be achieved by increasing income. Improving financial management or reducing or easing the financial choices that have to be made on a daily basis may be more promising. For example, it may be beneficial to support people dealing with poverty via coaching and concrete actions such as debt management. By reducing stress associated with a tight budget, cognitive bandwidth may be freed for other cognitive tasks such as self-control. At the same time, it may be worthwhile to decrease the level of self-control necessary for behaving healthily by making the healthy choice the easy one in a more facilitating social and physical environment.

Future research is necessary to further disentangle the cognitive pathways between income, financial strain and health behaviours. Related mechanisms, via stress, coping strategies and locus of control may play an important role as well. Furthermore, research that includes chronic financial stress may provide important insights in how stress can alter psychological variables such as self-control and affect regulation.

## Conclusion

Great financial strain is consistently associated with unhealthy behaviours, independent of income. Low self-control is also strongly associated with unhealthy behaviours and partly mediates between financial strain and unhealthy behaviour.

## Funding

This work was supported by a grant from the Netherlands Organization for Health Research and Development (grant number 200500005). The work has been presented at the European Public Health Conference in Vienna on November 10, 2016.

*Conflicts of interest*: None declared.

**Key points**

- Theory suggests that dealing with scarcity takes up cognitive bandwidth, which may impede a healthy lifestyle via depleted self-control.
- Perceived scarcity of money (financial strain) is more important for health behaviours than income.
- Impeded self-control only partially explains the association between financial strain and unhealthy behaviours.
- Interventions that relieve financial strain may improve health behaviour.
